# Effects of immersive virtual reality therapy on intravenous patient-controlled sedation during orthopaedic surgery under regional anesthesia: A randomized controlled trial

**DOI:** 10.1371/journal.pone.0229320

**Published:** 2020-02-24

**Authors:** Mark Y. Huang, Simon Scharf, Peter Y. Chan

**Affiliations:** 1 Department of Anaesthesia and Acute Pain Medicine, St Vincent’s Hospital Melbourne, Fitzroy, Victoria, Australia; 2 Department of Critical Care Medicine, St Vincent’s Hospital Melbourne, Fitzroy, Victoria, Australia; Universitatsklinikum Jena, GERMANY

## Abstract

**Background:**

Immersive virtual reality (IVR) is a form of distraction therapy that has shown potential as an analgesia and sedation sparing agent. This study assessed the effect of IVR on the self-administered sedation requirements of patients undergoing joint replacement surgery under regional anesthesia in a single center.

**Methods and findings:**

This study was a single**-**center, randomized control trial at St Vincent’s Hospital in Melbourne, Australia. Fifty patients undergoing elective total knee and total hip arthroplasty were randomized to IVR and Propofol patient-controlled sedation (PCS) or propofol PCS alone. The primary outcome measure was intra-operative propofol use. Secondary outcomes included pattern of propofol use over time, use of adjunct analgesia, unmet propofol demand, and patient satisfaction survey scores. Of 50 total patients, 25 received IVR in conjunction with PCS, and 25 received PCS alone. All patients received adjunct analgesia from the treating Anesthesiologist. Median propofol use/hour over the entire procedure in the control group was 40 (11.1, 93.9) mg/hour compared with 45 (0, 94.7) mg/hour in the IVR group (p = 0.90). There were no differences in patterns of propofol use over the course of each procedure. Adjusting for various baseline characteristics did not change the results. Postoperative satisfaction scores were equivalent in both groups. The VR intervention was well tolerated by all patients, with no report of major side effects. Key limitations were relatively small sample size, the non-blinded nature of the study, and use of adjunct analgesia.

**Conclusions:**

In patients receiving joint replacement surgery under regional anesthesia with PCS, IVR was well tolerated but did not decrease the overall sedation requirement.

## Introduction

Immersive virtual reality (IVR) is a form of distraction therapy, used as early as 2003 as an analgesia and sedation sparing agent [1], and has shown potential in the management of wound care, physical therapy, and anxiety-provoking procedures [2–5]. A typical IVR setup utilizes a head-mounted display (HMD) projecting an interactive, computer-generated environment with accompanying auditory stimuli, enhanced by head-tracking technology.

A pilot study of IVR using the Oculus Rift Virtual Reality HMD (Oculus Inc, California, USA) as an adjunct during joint surgery under regional anesthesia showed promise as a sedation sparing agent [6]. This study demonstrated a statistically significant reduction in sedation requirements, with average propofol use of 155mg/hour and average total use of 260mg in non-IVR patients, compared with average use of 63 mg/hr and average total use of 102mg in IVR patients [6]. These findings were limited by small numbers of participants, as well as the absence of blinding, which allowed selection bias and created the potential for anesthesiologists to unwittingly administer less propofol to the IVR group. Nonetheless, the study demonstrated the potential for a sedation sparing effect and provided the basis for a larger, well-designed, adequately powered study.

There have been several other studies that demonstrate a sedation or analgesic sparing effect of IVR [7–11]. Further efforts have also been made to commercialize IVR, with little high-quality evidence of effectiveness [12]. These projects suffer from the same limitations [6], namely small numbers and a heterogeneous population, poor blinding, and little bias control [13].

Patient controlled sedation (PCS) has been reported as early as 2013 to confer benefits during regional anesthesia [14], specifically allowing patients to control their own depth of sedation to ensure comfort without increasing complications. Ekin et al. reported the successful use of PCS during joint replacement surgery using a 400 mcg/kg bolus and a 5-minute lockout, demonstrating a mean usage of 133 mg of propofol over the duration of a procedure in 30 patients [14]. A recent review demonstrated the safety and efficacy of PCS, with a reduced risk of rescue interventions for sedation-related adverse events [15].

The current study aimed to address the limitations of recent studies, and better assess the potential sedation-sparing effect of IVR by combining it with PCS. Providing patients with control over sedation would theoretically minimize bias created by anesthesiologist-initiated sedation. It was hypothesized that IVR would reduce patient sedative requirement, lower patient anxiety, and maintain equivalent or greater patient satisfaction.

## Materials and methods

This study was a prospective, randomized controlled trial with a two arm parallel group design conducted at St Vincent’s Hospital Melbourne, Australia, conducted between 10 February and 2 June 2016. It was approved by the St Vincent’s Hospital Research and Ethics Committee (HREC Approval LRR 142/15) on 21 October 2015. Written and informed consent was obtained from all participants. This manuscript adheres to the Equator guidelines of the Consolidated Standards of Reporting Trials [16]. This trial was registered with the Australian New Zealand Clinical Trials Registry (http://www.ANZCTR.org.au/ACTRN12618001296224.aspx, Principal Investigator Dr. Peter Chan, date of registration 30/7/2018). Registration with the ANZCTR was delayed due to an administrative error, however, the protocol was not altered prior to registration, with no changes to the protocol after trial commencement. The authors confirm that all ongoing and related trials for this intervention are registered.

All patients undergoing elective knee or hip joint replacement surgery under regional anesthesia were eligible for enrolment. Inclusion criteria were English-speaking patients, 18 years of age and over, with no significant cardiovascular or respiratory disease. Exclusion criteria were patients receiving general anesthesia, cognitive impairment preventing the use of subjective outcome surveys, visual or hearing impairment and non-English speaking patients. Enrolled patients were randomized to the IVR or control group by computer-generated randomization in Microsoft Excel 2016 (Microsoft Corporation, Washington USA) with the process blocked every 10 patients to ensure 5 in each arm.

After insertion of an intravenous cannula and attachment of a crystalloid infusion, regional anesthesia for all patients was performed by the anesthesiologist. All patients received a spinal block, performed in the upright position. Skin numbing was achieved with 1% lidocaine, after which a 25g Whitacre needle was advanced at the L2-L4 interspace. After positive identification with clear flow of cerebrospinal fluid, between 2.8 and 3.4 ml of 0.5% Bupivacaine was injected into the subarachnoid space and the patient laid supine. For knee replacements, a popliteal nerve block was administered using 20ml of 0.2% ropivicaine and a femoral nerve catheter was inserted, with the patient receiving 20ml of 0.375% ropivicaine. Sensory and motor block was determined by pinprick and by attempting to elicit spontaneous movement of lower limbs.

At the discretion of the anesthesiologist, some patients also received either midazolam or fentanyl in this pre-operative period, either to tolerate the anesthetic procedure or to manage pre-operative anxiety. All patients received 5-minutely blood pressure, and continuous heart rate and oxygen saturation monitoring via a Philips MX800 Monitor (Koninklijke Philips, Netherlands) throughout, before, and during the procedure.

The IVR group received IVR using one of two available HMD setups depending on availability, with both including noise-cancelling headphones. The control group received no distraction and did not wear any HMD or headphones. Both groups were able to control their intra-operative sedation using propofol via patient-controlled sedation (PCS), consisting of an Alaris pump and PCA module (BD, Franklin Lakes, New Jersey, USA), with instruction to use it whenever they felt too aware or anxious. Each press of the PCS supplied a propofol bolus of 400mcg/kg Ideal Body Weight with a 5-minute lockout period. To prevent unwanted hypotension documented in previous studies [14] and on the advice from anesthesiologists reviewing the study protocol, an upper limit of 30mg per bolus was put in place as an additional safeguard. Anesthesiologists were advised that PCS should be the primary form of sedation but were not limited in giving additional sedation or analgesia if required in their clinical judgement. PCS and IVR if allocated were initiated in the operating room following spinal anesthetic, transfer to operating table and patient positioning, but before the beginning of the surgery. PCS and IVR were ceased once the procedure was complete.

The primary outcome was intra-operative propofol use, with the amount used and procedure duration recorded from the PCS machine after surgery. Secondary outcome measures included pattern of propofol over each hour, the amount of adjuvant midazolam or fentanyl used before the case, the overall unmet propofol demand, and postoperative patient satisfaction scores. There were no changes to trial outcomes after the trial commenced.

Patient experience was assessed using a modified Quality of Recovery Survey (QoR-40) [17]. The QoR-40 includes questions regarding patient comfort, emotional state, symptoms and pain rated on a 1–5 scale, where 1 means “never” and 5 means “all the time”. A modified version of the QoR-40 was used (S1 Appendix), employed before and after surgery. In addition, subjects were asked if they were satisfied with their experience, and if they would be willing to undergo future invasive surgeries using IVR.

### IVR simulation used

The IVR environment was provided by either a Samsung Gear VR HMD (Samsung, Korea) or the Oculus Rift Development Kit 2 (Oculus Inc, California USA) HMDs. Both HMDs feature a large field of view and high-quality display; the Gear VR with a 96-degree field of view and 1280 x 1440 pixel resolution per eye; and the DK2 with a 100-degree field of view and 960 x 1080 pixel resolution per eye. In addition, both HMDs block the user’s view of their immediate environment.

The Gear VR setup was powered by a Samsung Galaxy S6 phone (Samsung, Korea), running the freely available IVR software EdenRiver v1.0 by Unello Design (Unello Design, Texas, USA). This software allowed subjects to float along a procedurally generated three-dimensional river. The DK2 setup was powered by an MSI GS60-2QE gaming laptop (MSI Inc, Taiwan) running a custom-designed version of the software “Iceland” by Gert-Jan Werburg at VergeVR Inc (VergeVR, Netherlands) [18]. This software was modelled after the University of Washington software Snow World [19] and allowed subjects to travel through landscapes of an Arctic tundra. Both software were chosen as they provided a continuous “ride” experience, taking the user through the virtual environment without any need for directing travel. Both setups included noise-cancelling headphones playing background sound from the IVR simulation and classical music from the Hush Collection Volume 13 by the Tasmanian Symphony Orchestra (Hush Foundation, Victoria, Australia) provided with permission by Dr. Catherine Crock, pediatrician at the Royal Children’s Hospital, Melbourne.

### Statistical analysis

Data were analyzed on intention to treat principle. Distribution of variables was assessed visually by normal probability plots and using Shapiro-Wilk test. Majority of variables showed a non-normal distribution, therefore all baseline characteristics are presented as median (interquartile range) and frequency (percentage). Fisher’s Exact test and the Mann Whitney U test were used for the comparison of categorical and continuous non-parametric variables between groups respectively. Primary outcome (intra-operative propofol use) was calculated as number of PCS presses times amount of propofol per press and reflected as both total propofol use and use per hour. Comparison of propofol use between treatment arms was performed using negative binomial regression using total duration surgery as an offset. Model fit was assessed by visual inspection of deviance and Pearson residuals. Results are presented as incidence rate ratio with 95% confidence intervals. Due to small sample size and the effect of possible imbalances in baseline covariates despite randomisation, additional analysis (using negative binomial regression) adjusting for baseline covariates were performed. The intra-operative propofol use (total and per hour) in per-protocol population (those who used IVR throughout their procedure) was also compared using the rank sum test, however, the primary analysis still followed the intention to treat principle. Change in QoR-40 score was calculated as the difference between post and pre- procedure with negative values indicating the decrease in score. Mann Whitney U test was used for comparison of scores between the treatment arms.

For all comparisons, a two-sided p-value of <0.05 was used for statistical significance. Statistical analysis was performed using Stata 15.1 (StataCorp. 2017. *Stata Statistical Software*: *Release 15*. College Station, TX: StataCorp LLC).

### Sample size

The pilot study trialing the Oculus Rift as an adjunct during joint surgery under regional anesthesia showed that the IVR group used 63 ± 21 mg/hour propofol (mean ± SEM) and the control group used 155 ± 45 mg/hour (mean ± SEM). Standard deviation for the IVR group was 63.09 mg (n = 9) and 141.79 mg (n = 10) for the control group. [6]. Given the differences in variances, Welch’s t-test was employed and demonstrated that to achieve a power of 80% with a difference in means of 91 mg/hour, a sample size of 22 in each group would be required. To allow for a potential 10% attrition rate, 25 patients per group were recruited. There were no interim analyses or stopping guidelines used.

## Results

**Fig 1:**
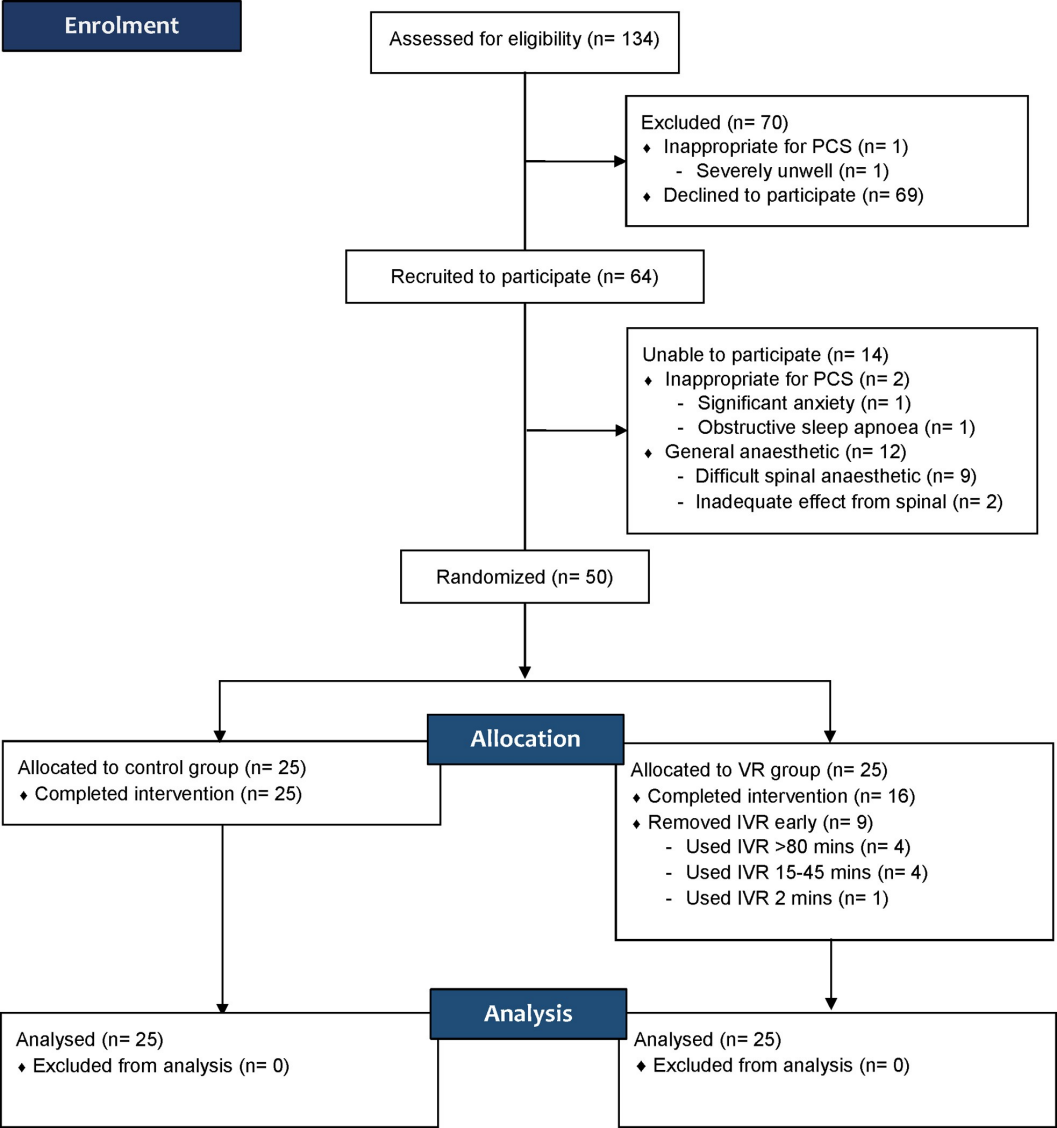
Patient recruitment, allocation and analysis.

A total of 134 patients were approached for this study between February and June 2016, of which 64 patients were enrolled to participate (Fig 1). Of these, 12 patients were later excluded as they required a general anesthetic for unforeseen reasons, including difficulty performing spinal anesthesia, inadequate effect from spinal anesthesia and failure to cease anticoagulant medication thus contraindicating spinal anesthesia. A further two patients were excluded after being deemed inappropriate for PCS by the treating anesthesiologist for other reasons. The remaining 50 patients were randomized to the control or IVR group, with 25 patients in each group. Of the 25 patients randomized to IVR, 16 (65%) utilized the HMD to the end of the procedure. Of the nine who removed the HMD early, four (16%) used it for more than 80 minutes, four (16%) used it for between 15–45 minutes, and one (4%) did not tolerate it at all. Reasons for early termination included discomfort, finding the VR software boring, and disliking the chosen software. The one patient who did not tolerate the IVR at all complained of worsening nausea that was present prior to the procedure. No other adverse outcomes were reported.

Baseline demographics and case characteristics of both groups are listed in Table 1. The median age in the IVR group was younger by five years, other baseline characteristics seem to be well balanced between 2 groups. The cases included 20 total knee joint replacements, 23 total hip joint replacements, five anterior total hip joint replacements, one hip hemiarthroplasty and one patellofemoral replacement. Case distribution, patient positioning and case duration was similar between the two groups. Pre-procedure Emotion-B score is included as it assesses patient anxiety, the scores of which were also similar between the two groups.

**Table 1:** Summary of demographic data from control and immersive virtual reality (IVR) groups.

	Control (N = 25)	IVR (N = 25)
**Gender**		
Female	12 (48)	13 (52)
Male	13 (52)	12 (48)
**Age (y)**	70 (64, 72)	65 (57, 68)
**Height (cm)**	164 (158, 172)	163.5 (157, 173)
**Weight (kg)**	80.5 (75, 98)	86 (76.5, 100)
**BMI (kg/m**^**2**^**)**	30.0 (27.5, 36.9)	33.0 (27.1, 36.9)
**Surgery duration (min)**	130 (115,140)	120 (105, 140)
**Procedure**		
Total knee replacement	10 (40)	11 (44)
Total hip replacement	15 (60)	14 (56)
**Position**		
Lateral	14 (56)	10 (40)
Supine	11 (44)	15 (60)
**Device**		
Samsung	-	13 (52)
Oculus	-	12 (48)
**Pre-procedure Emotion-B score**	7 (5, 8)	7 (6, 9)

Presented are N (%) or median (interquartile range).

**Fig 2:**
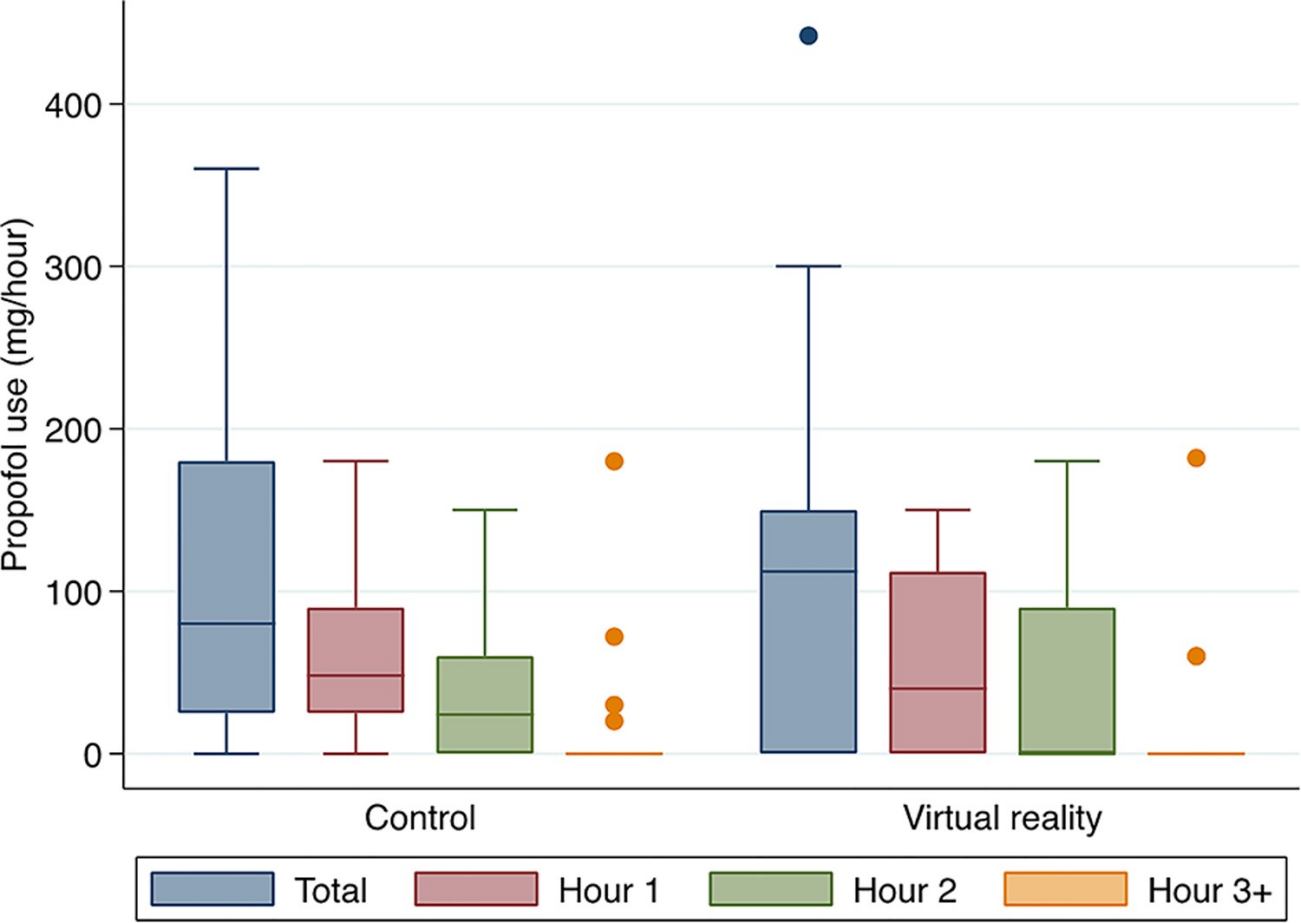
Propofol use and distribution over time.

A summary of intra-operative propofol use can be seen in Table 2. Median propofol use per hour over the entire procedure in the control group was 40 (11.1, 93.9) mg/hour compared with 45 (0, 95) mg/hour in the IVR group (p = 0.90). The total median use in the control and IVR groups was 80 (25, 180) mg and 112 (0, 150) mg respectively (p = 0.88). The minimum propofol used was 0 mg (12/50 patients) and the maximum propofol used was 442mg (1/50 patients). There was no significant difference in propofol use between the control and intervention arms at any time point. This is illustrated in Fig 2. The median number of unmet requests over the course of the entire case was 0 (IQR 0, 3) in control and 0 (IQR 0, 4) intervention arms (p = 0.61).

**Table 2:** Summary of intra-operative propofol use from control and immersive virtual reality (IVR) groups.

	Control (N = 25)	IVR (N = 25)	p
**Propofol use**			
Use per hour (mg/hr)	40 (11.1, 93.9)	45 (0, 94.7)	0.90
Hour 1 (mg)	48 (25, 90)	40 (0, 112)	0.64
Hour 2 (mg)	24 (0, 60)	0 (0, 90)	0.80
Hour 3+ (mg)	0 (0, 0)	0 (0, 0)	0.74
Total (mg)	80 (25, 180)	112 (0, 150)	0.88
**Met propofol requests**			
Hour 1	2 (1, 4)	2 (0,4)	0.41
Hour 2	1 (1, 2)	0 (0, 3)	0.83
Hour 3+	0 (0, 0)	0 (0, 0)	0.76
Total	3 (1, 7)	4 (0, 6)	0.66
**Unmet propofol requests**			
Hour 1	0 (0, 1)	0 (0, 2)	0.52
Hour 2	0 (0, 0)	0 (0, 0)	0.65
Hour 3+	0 (0, 0.)	0 (0, 0)	0.33
Total	0 (0, 3)	0 (0, 4)	0.61

Presented are median (interquartile range).

There was no significant difference in intra-operative propofol use (taking into account the duration of surgery) between IVR and control group (incidence rate ratio (IRR) 0.97 (95% CI 0.38, 2.45), p = 0.94). Adjusting for various baseline characteristics did not change the results (Table 3). Largest effect was observed after adjusting for the type of device used, when on average the rate of propofol used was for 70% lower in IVR group compared to control (95% CI from 96% smaller to 2.5 times larger, p = 0.26).

**Table 3:** Unadjusted and adjusted effect of virtual reality on propofol use.

	Incidence rate ratio (95% CI)	p
**Unadjusted**	0.97 (0.38, 2.45)	0.94
**Adjusted for age**	0.98 (0.38, 2.53)	0.97
**Adjusted for gender**	1 (0.38, 2.65)	0.99
**Adjusted for BMI**	0.94 (0.37, 2.39)	0.90
**Adjusted for type of procedure**	0.96 (0.38, 2.42)	0.94
**Adjusted for position**	0.96 (0.38, 2.44)	0.94
**Adjusted for device used**	0.3 (0.04, 2.5)	0.26

There was a total of 9 patients who removed the HMD early. After the exclusion of these patients, propofol use remained similar (22.1 mg/hour (IQR 0, 94.5) in IVR group and 40 mg/hour (IQR 11.1, 93.9) in control group, p = 0.37). The total propofol use was smaller, however there were no differences between groups (35 (IQR 0, 165) mg in IVR group and 80 (IQR 25, 180) mg in control group, p = 0.36). Note that the primary analysis was still performed following the intention to treat principle.

Midazolam and fentanyl administration during the procedures are listed in Table 4. The median fentanyl use in the IVR group and the control group over the course of the entire procedure was 37.5 (0, 50) mcg and 0 (0, 50) mcg respectively (p = 0.22), while the median midazolam use over the procedure was 2.3 (2, 3) mg and 1.5 (1, 2) mg respectively (p = 0.10).

**Table 4:** Summary of midazolam and fentanyl use before and during procedure.

	Control	IVR	p
**Midazolam (units)**			
Pre-procedure	1.5 (1, 2)	2.3 (2, 3)	0.042
During procedure	0 (0, 0)	0 (0, 0)	0.25
Total	2 (1, 3)	2.3 (2, 3)	0.10
**Fentanyl (units)**			
Pre-procedure	0 (0, 50)	37.5 (0, 50)	0.28
During procedure	0 (0, 0)	0 (0, 0)	0.75
Total	0 (0, 50)	37.5 (0, 50)	0.22

Presented are median (interquartile range).

Both groups reported an overall decrease in QoR-40 score with an overall median decrease of 7 points. Both groups also reported a considerable decrease in pain score (median decrease of 6 in control and 5 in IVR group) (Table 5). There was, however, no significant difference between the groups in any component of QoR-40 score, with the exception of confusion score where 3 patients in control group reported a decrease, while no patient in IVR group reported any change (p = 0.08).

**Table 5:** Median (IQR) change in QoR-40 scores from before and after procedure.

	Control	IVR	p
Comfort Score Change	0 (-2, 2)	0 (-1, 2)	0.46
Emotion Score Change	1 (0, 2)	0 (0, 2)	0.15
Symptom Score Change	0 (-1, 0)	0 (-1, 0)	0.80
Emotion (B) Score Change	-2 (-3, 0)	-2 (-3.5, 0)	0.61
Confusion Score Change	0 (0, 0)	0 (0,0)	0.08
Pain Score Change	-6 (-9, -4)	-5 (-8, -3)	0.60
Overall Score Change	-7 (-10, -4)	-7 (-10.5, 1)	0.82

Additional questions regarding patient satisfaction found that all patients in both treatment groups were satisfied with their experience. All but one patient using IVR distraction would use it again. The one patient who would not use IVR again found use of the HMD to be “claustrophobic” but still reported satisfaction with trying the experience. The majority of comments from patients were positive, including “It helped take my mind off the surgery”, “It was very relaxing”, “I felt like I was in a beautiful place” and “I really enjoyed that”. Other comments indicated patients wanted more choice in IVR experience, “It was fantastic, though a bit boring” and “It was a little repetitive, I wanted to try more”.

No major adverse outcomes were reported that were directly attributable to the IVR. There was one episode of nausea that was pre-existing before institution of IVR that necessitated removal of the HMD. There were no documented episodes of hypotension, bradycardia, or injection site pain associated with the administration of PCS.

## Discussion

The current study aimed to address the limitations of recent IVR studies, by attempting to eliminate the Anesthesiologist as a source of potential bias by combining IVR with PCS. There was no significant difference in patient-controlled intra-operative propofol use during joint replacement surgery under regional anesthesia when using IVR compared with the control group receiving no IVR, reflected in the total dose required per case, the amount per hour, and the amount administered in any particular hour. As propofol was delivered as a weight-based dose, the number of demands per patient was also examined, and there was no difference in demands between the two groups. In both groups, the median unmet demand was zero, suggesting that the PCS was adequate to maintain patient comfort. IVR did not affect the pattern of propofol use, with no significant difference observed in propofol use during the first, second, and third hours of the procedures. There was no difference in the administration of anxiolytic or analgesic agents between the two groups. QoR-40 scores found no difference in patient satisfaction between treatment groups.

In our previous study, where anesthesiologists controlled the amount of sedation given, the mean propofol use was 155 ± 45 mg/hr in the routine care group [6]. This compares to 41 (2.3, 94.3) mg/hr in the current study, where the sedation was patient controlled. Ekin et al [14] equally reported lower doses of self-administered propofol in their study compared to our pilot study. As a whole, this suggests that the lower sedation doses in the IVR group seen in the pilot study was not the result of IVR, but a lower overall sedation requirement than what was administered as a routine by the anesthesiologist.

Use of IVR was overall well-received, with all patients reporting satisfaction with the experience, as well as many positive comments. Quality of Recovery was the same between the two groups, indicating that it provided at least an equal experience. Despite this, nine of the 25 patients randomized to the IVR arm removed the HMD early, suggesting that not every patient remains engaged with the IVR for the entire duration of the procedure. The inclusion of these patients in the analysis may have reduced the ability to discern a treatment effect. As such, the mean propofol dose was also calculated amongst only the patients who used IVR for the entirety of the procedure. The mean propofol use per hour and mean total propofol use in this cohort, while less than that when all patients were included, was still nonsignificant.

Given 36% of patients chose to remove their HMD early, the addition of more engaging content may be needed in order to demonstrate a potential effect. While the chosen software was generally well-received, some patients became bored and sought more engaging options. A limitation of both Iceland and Eden River was that they could become repetitive, with a number of patients commenting that increasing the selection of content would have improved their experience. At the time of this study, the selection of IVR software was limited, as many options were often still in early development, or were games requiring a gamepad or significant head movements, neither of which are appropriate during a surgical procedure. A number of IVR studies have utilized the Snow World software by the University of Washington [19], which allows some interaction with the virtual environment in the form of throwing of virtual snowballs at passing objects. This may allow greater engagement with the virtual environment, with greater engagement postulated to have a greater analgesic effect [20]. Software that may be able to achieve greater engagement in the operating theatre environment would likely be a ride experience similar to those chosen for the current study, but with a choice of environments and a multiple ways to interact with the environment, such as through small head movements and potentially a simple gamepad for the patient, where a single button would be enough to pick up and throw objects.

There are a number of other limitations noted in this study that may have affected detection of any treatment effect. The non-blinded nature of the study could lead to differences in the way the treating anesthesiologist administers additional sedation such as midazolam and fentanyl. There was no significant difference observed in total midazolam and fentanyl use between the two groups, however, their effect on PCS requirements is difficult to quantify. While blinding the use of an HMD to then anesthesiologist is not practical, future studies should aim to standardize the use of sedation other than PCS to minimize variability in practice between anesthesiologists and its likely confounding of the primary outcome. Inclusion of patients in both supine and lateral position may have also confounded results, as these two subgroups are likely to have different sedation requirements, with the lateral positioning being more uncomfortable. It should also be noted that almost half of the patients approached for this study declined, preferring to have analgesia and sedation managed by the anesthesiologist without IVR. These patients tended to cite anxiety over the upcoming procedure as a reason for declining to participate. It is unknown how these more anxious patients might have responded to IVR compared to the cohort that agreed to the trial, or how their inclusion might have affected postoperative satisfaction scores.

This study does not support the hypothesis that IVR confers a sedation sparing effect on patients receiving joint replacement surgery under regional anesthesia. It does, however, demonstrate that it is feasible to implement IVR without much difficulty in a busy operating theatre. As with the trial of any new intervention with a small sample size, care must be made not to overinterpret preliminary findings, and to be wary of potential sources of bias. There appear to be many factors that have greater influence on sedation requirements over the effect of IVR, including the use of adjunctive pharmacological agents, the type of surgery, and pre-existing patient anxiety.

An IVR treatment benefit may potentially exist by selecting patients with higher sedation requirements, including younger patients, those with high pre-procedure anxiety, and those expected to have significant discomfort during a procedure due to positioning or other factors. Testing this hypothesis, however, would most likely require more personalized and engaging content in order to keep patients engaged with IVR for the duration of the procedure. A much larger study targeting this smaller subset of individuals, while also controlling for confounders such as patient positioning and the use of adjunctive pharmacological agents, would also likely be necessary to show any benefit of IVR.

## Supporting information

S1 Checklist CONSORT 2010 checklist of information to include when reporting a randomised trial*. (DOC)

S2 Checklist Low risk research application form. (DOCX)

S1 Data (XLSX)

S1 Appendix Datasheet with modified QoR-40. (PDF)
