# Supplementary material for: Effects of immersive virtual reality therapy on intravenous patient-controlled sedation during orthopaedic surgery under regional anesthesia: A randomized controlled trial
Source: PLoS One. 2020 Feb 24;15(2):e0229320. doi: 10.1371/journal.pone.0229320 (PMC7039521; doi:10.1371/journal.pone.0229320)
Supplement: S2 Checklist — (DOCX) [file pone.0229320.s002.docx]

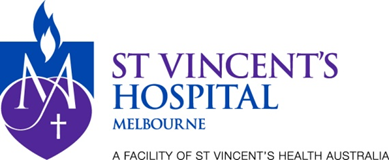


LOW RISK RESEARCH APPLICATION FORM

CHECKLIST

If you answer YES to any of the following items, the research will not be considered as Low Risk Research. Please discontinue using this form and apply to Human Research Ethics Committee-A.

Vulnerable Groups

| Women who are pregnant |  |  |
| --- | --- | --- |
| Children or young people under the age of 18 |  |  |
| Persons with cognitive impairment, an intellectual disability or a mental illness |  |  |
| Persons highly dependent on medical care |  |  |
| Persons incompetent to consent for themselves |  |  |
| People involved in illegal activities |  |  |
| Prisoners or people on parole |  |  |
| Research specifically recruiting ATSI (Aboriginal and / or Torres Strait Islander people) |  |  |
| Persons considered to be vulnerable in the context of this research project |  |  |
|  |  |  |
| Determination of Risk |  |  |
| Establishment of a databank for possible use in future research |  |  |
| Interventions and therapies including clinical and non-clinical trials and innovations as defined in Chapter 3.3 of the National Statement on Ethical Conduct in Human Research |  |  |
| Human genetic research or gene technology |  |  |
| Derivation or use of human stem cells |  |  |
| Causing discomfort to participants beyond normal levels of inconvenience |  |  |
| Deception of participants, concealment or covert observation |  |  |
| Examining potentially sensitive or contentious issues |  |  |
| Radioactive substances / ionising radiation e.g. X-rays, DEXA |  |  |
| Assisted reproductive technology |  |  |
| Xenotransplantation |  |  |
| Toxins / Mutagens / Teratogens / Carcinogens |  |  |
| The collection, use or disclosure of individually identifiable or re-identifiable information without the consent of the individual whose information it is (or their legal guardian) OR a waiver of consent from a HREC |  |  |
| The research poses risks for patients beyond those of their routine care |  |  |

If you answered *YES* to any of the above mentioned items, the research is considered *NOT* to be Low Risk Research. Please discontinue using this form and apply to the Human Research Ethics Committee-A as specified on the Research Governance Unit website at http://www.svhm.org.au/RESEARCH/GOVERNANCE/Pages/governance.aspx

If you answered *NO* to all of the questions above, please complete the remainder of the form

SECTION A: PROJECT OVERVIEW

- 1. Full project title

| The Use of Virtual Reality (VR) Environments in a Clinical Setting |
| --- |

1.2 Lay summary of the project

| We have recently been funded for a pilot study to assess the safety of using Oculus Rift Virtual Reality Goggles in a clinical setting with the ultimate goal of testing their effectiveness in reducing delirium in an ICU population. Oculus Rift goggles are virtual reality headsets which create a 3D video image that moves with patient head movements. They simulate virtual environments that are immersive and that create so-called "illusion of presence," or the perception of a virtual object as existing in the physical world. These goggles have previously been trialled on children during burns dressing changes with very good effect.  As a portion of the original pilot study assessing the efficacy of this intervention, a pilot study was performed on well patients awaiting discharge from the High Dependency Unit as well as patients receiving joint replacement surgery under regional anaesthesia in the operating theatre.  The initial pilot study ( LRR 039/15) aimed to test the Immersive Virtual Reality (IVR) therapy on approximately 25 patients in the ICU who were clinically well, bed blocked, and without significant residual medical problems, quantified by having a Sequential Organ Failure Assessment Score (SOFA) of under 5. In addition, we planned on testing the goggles on 25 otherwise well patients undergoing regional anaesthetic and who are fully awake for total knee and total hip replacements in the operating theatre. A control group of 25 joint replacement patients who would not receive the therapy were also to be asked to complete a survey.  The pilot study was performed in the anaesthetic department from May to July 2015 on 10 patients receiving regional anaesthesia for knee, hip, and ankle surgery. The initial plan was to perform this study on 25 patients, but it became readily apparent that there was a significant reduction in the amount of sedation being used on patients with VR therapy compared to patients receiving standard, routine care. However, given the anaesthetist was controlling the amount of sedation being delivered, it was not possible to determine if this effect was as a result of a conscious decision on the part of the anaesthetist to use less sedation, or a true reduction in need as a result of IVR therapy.  As a result, we have enlisted the help of a scholarly selective student from the University of Melbourne to test the new hypothesis: In patients receiving regional anaesthesia for orthopedic surgery, does the use of patient controlled sedation (PCS) via propofol TIVA combined with IVR therapy result in a better subjective experience and less propofol used compared to PCS alone?  This trial is therefore an offshoot based on the positive preliminary data gained from one aspect of the pilot study.  Each patient will receive and complete a questionnaire that has been validated by the Department of Anaesthesia (Patient Survey QoR-40) assessing their comfort, emotions and pain scores that will be completed post cessation of treatment. Vital signs will also be collected during the intervention. Their scores will be compared with age-matched controls.  Patients will be de-identified, but additional demographic data, as well as past medical data will be collected.  The primary outcome measure will be the amount of sedation used during the procedure. Secondary outcome measures include the use of adjunctive sedation, the presence of side effects including nausea and motion sickness, and subjective patient satisfaction as quantified by the QoR-40 score. |
| --- |

Briefly describe the project using plain everyday language and describing key aspects of the project i.e. who will be participating, what information will be collected and by what means, types of analysis to be performed etc.

1.3 Relationship to other projects

Is the project:

If the project is a sub-component of, or in some other way related to, a previously approved project, provide project numbers for the other project(s). Also indicate which HREC(s) approved the other project(s).

| LRR 039/15 |
| --- |

1.4 Multi-site projects

Is the project a multi-site project? That is, does the project involve recruitment of participants at more than one site and/or collection of information from more than one organisation?

Does the project have to be reviewed by other HRECs?

Name **all** **Australian HRECs** to which this project has been or will be submitted including the SVHM LRR Sub-committee included in the table provided over page. For each HREC, list all Australian sites involved in this project that are covered by the application to that HREC. If the number of sites for a particular HREC is very large (or unknown), such that listing individual sites is not feasible, indicate the number of sites covered by that HREC (e.g. 50 primary schools or 20 out of 60 child care centres, etc.). Indicate the status of the application to other HRECs.

| HREC or other review body  *(must include STV Low Risk Research Sub-committee)* | Site  *(must include all sites for which STV is responsible*) | Status of application  (*e.g. not yet applied/approved/ rejected/pending*) |
| --- | --- | --- |
| SVHM Low-Risk Research Subcommittee | St Vincent's Hospital Fitzroy | Pending |

SECTION B: RESEARCHERS AND CONTACT INFORMATION

1.5 List all researchers involved in this project

*Principal Researcher:*

| Title and Name | Dr. Peter Chan |
| --- | --- |
| Department | Department of Critical Care Medicine |
| Institution | St Vincent's Hospital |
| Mailing address | ICU 41 Victoria Pde Fitzroy, 3065 |
| Describe what this researcher will do in the context of this project | Principal investigator - data collection and analysis, running simulation, liasing with Dept. of Anaesthesia |
| Include a brief summary of relevant experience for this project | ICU Registrar, significant publication history. Funding approved by Research Endowment Fund and Catalyst Innovation Fund |
| Phone | 0422525247 |
| Email | Peter.chan@svhm.org.au |

*Copy this table and repeat for each* ***Associate Researcher****:*

| Title and Name | Dr. Simon Scharf |
| --- | --- |
| Department | Department of Anaesthesia |
| Institution | St Vincent's Hospital |
| Mailing address | 41 Victoria Pde Fitzroy, 3065 |
| Describe what this researcher will do in the context of this project | Coordinate running simulation in the operating theatres during total knee and total hip replacement surgery |
| Include a brief summary of relevant experience for this project | Deputy Head of Acute Pain services with research interest in novel pain management modalities |
| Phone | 9288 4253 |
| Email | Simon.scharf@svha.org.au |

Copy this table and repeat for each Associate Researcher:

| Title and Name | Dr. Rowan Thomas |
| --- | --- |
| Department | Department of Anaesthesia |
| Institution | St Vincent's Hospital |
| Mailing address | 41 Victoria Pde Fitzroy, 3065 |
| Describe what this researcher will do in the context of this project | Coordinate running simulation in the operating theatres during total knee and total hip replacement surgery |
| Include a brief summary of relevant experience for this project | Deputy Director of Department of Anaesthesia with research interest in novel pain management modalities |
| Phone | 9288 4253 |
| Email | Rowan.thomas@svha.org.au |

*Copy this table and repeat for each* ***Student Researcher****:*

| Title and Name | Mr. Mark Huang |
| --- | --- |
| Department | Department of Anaesthesia and Clinical Pain Medicine |
| Institution | St Vincent's Hospital Clinical School, University of Melbourne |
| Mailing address | 41 Victoria Parade, Fitzroy VIC 3065 |
| Degree/Course | MD |
| Describe what this researcher will do in the context of this project | Run day to day simulation under the supervision of attending anaesthetist |
| Include a brief summary of relevant experience for this project | None. This project is to be completed as a portion of his mandatory Scholarly Selective Program |
| Phone | 0430 177 661 |
| Email | m.huang@student.unimelb.edu.au |

1.6 Training

Will any of the researchers require extra training to enable their participation in this project?

If *Yes*, please complete the following table:

| **Researcher** | **Training required** | **Who will provide training?** |
| --- | --- | --- |
| Mark Huang | Simulation, experimental design, ethics, statistics | Peter Chan |

SECTION C: PROJECT DETAILS

1.7 Anticipated duration of project: 1 year

1.8 Anticipated commencement date at this site: 30/01/2016

1.9 Anticipated completion date at this site: 15/2/2017

1.10 Project proposal

You may type (or “paste”) your detailed proposal directly into the text box below (including date and version number) and/or you may attach pre-printed document(s). Attachments should include brochures/pamphlets, questionnaires or surveys and any other relevant documents. Please ensure that all attachments are page numbered throughout and given a version number and date where appropriate.

The type of information that should be included in the detailed proposal includes Literature review; Rationale of project; Primary hypotheses and/or research questions (if applicable); Aims; Methodology or Project Design including description of research procedures, surveys and questionnaires, recruitment strategies and any other relevant information; Inclusion/Exclusion criteria (if applicable); Randomisation procedures (if applicable); and Statistical or other analyses.

| Version Date: 20/07/2015 | Version Number: 1.03 |
| --- | --- |
| Project Title: The use of Immersive Virtual Reality Therapy as an Adjunct to Regional Anaesthesia in Orthopedic Surgery  Principal investigator: Peter Chan, MBBS (Hons) BSc. (Hons)  Background, Aims, Hypothesis:  The use of Virtual Reality in medicine has largely been confined to surgical simulation and psychiatric study. There has been some promising use of VR as an adjunct in chronic pain management (1), exposure therapy for PTSD(2), treatment of phantom limb pain (3), and stroke rehabilitation(4).  Recent advances in technology have paved the way for point-of-care VR headsets that can be used at the patient bedside. Specifically, the Oculus Rift is an inexpensive ($400) wide field of view 3D VR headset that projects video and rendered graphics into two independent lenses, creating the perception of a virtual object as existing in the physical world, or so-called “illusion of presence(5).” The current model is the size of a small pair of ski goggles, weighs 440g, and is maintained on the head with simple elastic straps. We have been recently experimenting with 360 degree video combined with virtual environments, projected through the glasses.  Regional anaesthesia in joint surgery is common practice, and offers several advantages to general anaesthetic, including spontaneous respiration, cough and sputum clearance, lower cost, and shorter hospital stay (6) Owing to the fact that patients are fully awake, however, and coupled with the noise or a busy operating theatre, patients are often given procedural sedation in addition to their regional anaesthetic. Procedural sedation provides anxiolysis, reduce postoperative recall, and better tolerance of the procedure. It has also been shown to reduce analgesic requirements, post-operative nausea, and vomiting (6). However, the use of sedation in an unprotected airway, also confers a small element of risk, not limited to aspiration, airway obstruction, and hemodynamic instability (6). Minimizing sedation while maintaining patient comfort would therefore have safety, satisfaction, and cost saving benefits. One way to minimize sedation and increase satisfaction and patient autonomy has been through the use of patient controlled sedation by way of a propofol PCA, used effectively in previous studies during joint replacement surgery with no adverse effects. (7)  As a pilot and feasibility study to test the efficacy of IVR and as a part of this initial ethics application, IVR was used in the operating theatre at St Vincent's Hospital Melbourne in patients receiving joint replacement surgery under regional anaesthetic (Figure A). It demonstrated not only the safety and efficacy of applying IVR while administering intravenous anaesthetic agents including propofol, fentanyl, and midazolam, but also that it is well tolerated and provides a comfortable and relaxing experience. Furthermore, IVR demonstrated equivalent satisfaction and pain scores in intraoperative patients, while receiving significantly (p<0.05) less procedural sedation than those without IVR without an increase in unpleasant side effects including nausea and vomiting.  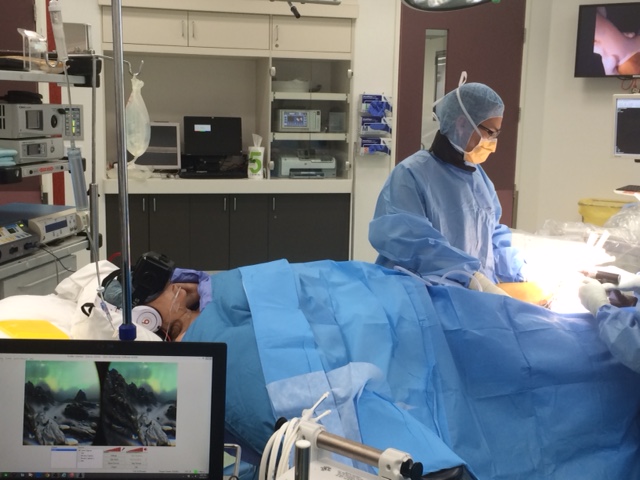  Figure A: IVR therapy on patient receiving knee replacement surgery under regional anaesthetic  Given the anaesthetist was in control of the amount of sedation being used, and he was not blinded to the administration of propofol, ethics for a secondary followup trial is being sought.  Aims: To implement a pilot feasibility trial VR environments on patients who can control their own level of sedation to assess tolerance, satisfaction and side effects. To establish whether the use of IVR therapy results in a reduction of sedation needed to maintain equivalent or better satisfaction scores compared to patients not receiving IVR therapy.  Hypothesis: We hypothesize that in patients receiving joint replacement surgery with regional anaesthesia and patient-controlled sedation pumps, IVR therapy will result in a lower amount of sedation needed, with equivalent or superior levels of patient satisfaction. Furthermore, we anticipate that IVR will be well tolerated and beneficial to patients, with subjective reduction in anxiety.  Methodology: All and pre-op total knee and total hip replacement patients will be informed of the study and will be given written information and be offered the chance to participate. If they agree to participate, recruitment will be done by the principal investigator, research nurse, or research assistant.  Inclusion criteria: English-speaking patients 18 years of age with and over no significant cardiovascular or respiratory disease.  Exclusion criteria: Patients receiving general anesthesia, cognitive impairment preventing the use of subjective outcome surveys, visual or hearing impairment and non-English speaking patients.  Baseline measures: Age, sex, EUC, BSL, diagnosis, current or previous employment, days in ICU, history of motion sickness or nausea, baseline heart rate and blood pressure, past medical history, current medications  Outcomes: Primary outcome: Total and mean amount of propofol sedation used during case. Secondary Outcomes: Presence of nausea, nausea resolved with antiemetic, presence of sleep during or after therapy, score on QoR-40 form before and after proecedure. Adjunctive sedation used.  Software simulations used: A custom designed version of freely available software called Iceland, designed by VergeVR inc. ([www.vergevr.com](http://www.vergevr.com)) will be used for the pilot study in addition to Edenriver by Unello design.. This will be coupled with music from the Tasmanian Symphony Orchestra, from the Hush Collection, borrowed with permission from Dr. Catherine Crock at the Royal Children's Hospital in Melbourne. This is the same setup and simulation that was used for the initial pilot study.  Randomisation and allocation concealment: Patients will be randomized to treatment or control group by computer randomization. 25 patients will be allocated to IVR and 25 patients to conventional therapy. Randomisation will be blocked to ensure that after 5 patients, equal numbers will be allocated to the 2 groups. Given the fact that wearing the goggles and headphones cannot be concealed, this study will be randomized but not blinded.  Administration of IVR: Based on our experience from the pilot study, the simulation is well tolerated for the duration of the surgery. Propofol TIVA via PCA will be applied with 400 microgram/kg boluses with a 5 minute lockout period. Routine anaesthetic observations, will ensure that oversedation does not occur. There will be a 30 mg limit to each bolus to prevent unwanted apnoea or hypotension.  Recruitment feasibility: Based on historical data, we anticipate approximately 10 patients per week will meet the inclusion criteria. Allowing for competing trials and non-consent, we believe it will be possible to recruit the 50 proposed patients over a 6 month period.  Statistical methods: Analysis will be conducted on an intention-to-treat basis. Differences in outcomes will be compared using the Student's t-test for normally distributed data and Wilcoxon rank-sum tests otherwise. Categorical outcomes will be assessed using Pearson's unconditional chi-squared test or Fisher's Exact test, as appropriate. For comparison of normally distributed data between more than 2 groups, ANOVA will be used.  Sample size calculation: As a trial of this nature has not been attempted before, a total of twenty-five patients have been chosen as a number that can be feasibly accomplished in six months, and will provide enough information with regards to standard deviation and treatment effect, that can be used to properly power future studies.  References: 1. [Hoffman HG](http://www.ncbi.nlm.nih.gov/pubmed?term=Hoffman%20HG%5BAuthor%5D&cauthor=true&cauthor_uid=21264690), [Chambers GT](http://www.ncbi.nlm.nih.gov/pubmed?term=Chambers%20GT%5BAuthor%5D&cauthor=true&cauthor_uid=21264690), [Meyer WJ](http://www.ncbi.nlm.nih.gov/pubmed?term=Meyer%20WJ%203rd%5BAuthor%5D&cauthor=true&cauthor_uid=21264690), [Arceneaux LL](http://www.ncbi.nlm.nih.gov/pubmed?term=Arceneaux%20LL%5BAuthor%5D&cauthor=true&cauthor_uid=21264690), [Russell WJ](http://www.ncbi.nlm.nih.gov/pubmed?term=Russell%20WJ%5BAuthor%5D&cauthor=true&cauthor_uid=21264690), [Seibel EJ](http://www.ncbi.nlm.nih.gov/pubmed?term=Seibel%20EJ%5BAuthor%5D&cauthor=true&cauthor_uid=21264690), [Richards TL](http://www.ncbi.nlm.nih.gov/pubmed?term=Richards%20TL%5BAuthor%5D&cauthor=true&cauthor_uid=21264690), [Sharar SR](http://www.ncbi.nlm.nih.gov/pubmed?term=Sharar%20SR%5BAuthor%5D&cauthor=true&cauthor_uid=21264690), [Patterson DR](http://www.ncbi.nlm.nih.gov/pubmed?term=Patterson%20DR%5BAuthor%5D&cauthor=true&cauthor_uid=21264690). Virtual reality as an adjunctive non-pharmacologic analgesic for acute burn pain during medical procedures. [Ann Behav Med.](http://www.ncbi.nlm.nih.gov/pubmed/21264690) 2011 Apr;41(2):183-91. doi: 10.1007/s12160-010-9248-7. 2. [Rizzo A](http://www.ncbi.nlm.nih.gov/pubmed?term=Rizzo%20A%5BAuthor%5D&cauthor=true&cauthor_uid=24732532), [Hartholt A](http://www.ncbi.nlm.nih.gov/pubmed?term=Hartholt%20A%5BAuthor%5D&cauthor=true&cauthor_uid=24732532), [Rothbaum B](http://www.ncbi.nlm.nih.gov/pubmed?term=Rothbaum%20B%5BAuthor%5D&cauthor=true&cauthor_uid=24732532), [Difede J](http://www.ncbi.nlm.nih.gov/pubmed?term=Difede%20J%5BAuthor%5D&cauthor=true&cauthor_uid=24732532), [Reist C](http://www.ncbi.nlm.nih.gov/pubmed?term=Reist%20C%5BAuthor%5D&cauthor=true&cauthor_uid=24732532), [Kwok D](http://www.ncbi.nlm.nih.gov/pubmed?term=Kwok%20D%5BAuthor%5D&cauthor=true&cauthor_uid=24732532), [Leeds A](http://www.ncbi.nlm.nih.gov/pubmed?term=Leeds%20A%5BAuthor%5D&cauthor=true&cauthor_uid=24732532), [Spitalnick J](http://www.ncbi.nlm.nih.gov/pubmed?term=Spitalnick%20J%5BAuthor%5D&cauthor=true&cauthor_uid=24732532), [Talbot T](http://www.ncbi.nlm.nih.gov/pubmed?term=Talbot%20T%5BAuthor%5D&cauthor=true&cauthor_uid=24732532), [Adamson T](http://www.ncbi.nlm.nih.gov/pubmed?term=Adamson%20T%5BAuthor%5D&cauthor=true&cauthor_uid=24732532), [Buckwalter JG](http://www.ncbi.nlm.nih.gov/pubmed?term=Buckwalter%20JG%5BAuthor%5D&cauthor=true&cauthor_uid=24732532)1. Expansion of a VR Exposure Therapy System for Combat-Related PTSD to Medics/Corpsman and Persons Following Military Sexual Trauma. [Stud Health Technol Inform.](http://www.ncbi.nlm.nih.gov/pubmed/?term=Stud+Health+Technol+Inform.+2014%3B196%3A332-8) 2014;196:332-8. 3. [Ortiz-Catalan M](http://www.ncbi.nlm.nih.gov/pubmed?term=Ortiz-Catalan%20M%5BAuthor%5D&cauthor=true&cauthor_uid=24616655), [Sander N](http://www.ncbi.nlm.nih.gov/pubmed?term=Sander%20N%5BAuthor%5D&cauthor=true&cauthor_uid=24616655), [Kristoffersen MB](http://www.ncbi.nlm.nih.gov/pubmed?term=Kristoffersen%20MB%5BAuthor%5D&cauthor=true&cauthor_uid=24616655), [Håkansson B](http://www.ncbi.nlm.nih.gov/pubmed?term=H%C3%A5kansson%20B%5BAuthor%5D&cauthor=true&cauthor_uid=24616655), [Brånemark R](http://www.ncbi.nlm.nih.gov/pubmed?term=Br%C3%A5nemark%20R%5BAuthor%5D&cauthor=true&cauthor_uid=24616655). Treatment of phantom limb pain (PLP) based on augmented reality and gaming controlled by myoelectric pattern recognition: a case study of a chronic PLP patient. [Front Neurosci.](http://www.ncbi.nlm.nih.gov/pubmed/?term=Front+Neurosci.+2014+Feb+25%3B8%3A24) 2014 Feb 25;8:24. doi: 10.3389/fnins.2014.00024. eCollection 2014. 4. [McEwen D](http://www.ncbi.nlm.nih.gov/pubmed?term=McEwen%20D%5BAuthor%5D&cauthor=true&cauthor_uid=24763929), [Taillon-Hobson A](http://www.ncbi.nlm.nih.gov/pubmed?term=Taillon-Hobson%20A%5BAuthor%5D&cauthor=true&cauthor_uid=24763929), [Bilodeau M](http://www.ncbi.nlm.nih.gov/pubmed?term=Bilodeau%20M%5BAuthor%5D&cauthor=true&cauthor_uid=24763929), [Sveistrup H](http://www.ncbi.nlm.nih.gov/pubmed?term=Sveistrup%20H%5BAuthor%5D&cauthor=true&cauthor_uid=24763929), [Finestone H](http://www.ncbi.nlm.nih.gov/pubmed?term=Finestone%20H%5BAuthor%5D&cauthor=true&cauthor_uid=24763929). Virtual reality exercise improves mobility after stroke: an inpatient randomized controlled trial. [Stroke.](http://www.ncbi.nlm.nih.gov/pubmed/?term=(Stroke.+2014+Jun%3B45(6)%3A1853-5) 2014 Jun;45(6):1853-5. doi: 10.1161/STROKEAHA.114.005362. Epub 2014 Apr 24. 5. [Hoffman HG](http://www.ncbi.nlm.nih.gov/pubmed?term=Hoffman%20HG%5BAuthor%5D&cauthor=true&cauthor_uid=12804024), [Richards T](http://www.ncbi.nlm.nih.gov/pubmed?term=Richards%20T%5BAuthor%5D&cauthor=true&cauthor_uid=12804024), [Coda B](http://www.ncbi.nlm.nih.gov/pubmed?term=Coda%20B%5BAuthor%5D&cauthor=true&cauthor_uid=12804024), [Richards A](http://www.ncbi.nlm.nih.gov/pubmed?term=Richards%20A%5BAuthor%5D&cauthor=true&cauthor_uid=12804024), [Sharar SR](http://www.ncbi.nlm.nih.gov/pubmed?term=Sharar%20SR%5BAuthor%5D&cauthor=true&cauthor_uid=12804024). The illusion of presence in immersive virtual reality during an fMRI brain scan. [Cyberpsychol Behav.](http://www.ncbi.nlm.nih.gov/pubmed/12804024) 2003 Apr;6(2):127-31.  [6](file:///E:\Chanvision\Phase%202\%206). [Höhener D](http://www.ncbi.nlm.nih.gov/pubmed/?term=H%C3%B6hener%20D%5BAuthor%5D&cauthor=true&cauthor_uid=18070783)1, [Blumenthal S](http://www.ncbi.nlm.nih.gov/pubmed/?term=Blumenthal%20S%5BAuthor%5D&cauthor=true&cauthor_uid=18070783), [Borgeat A](http://www.ncbi.nlm.nih.gov/pubmed/?term=Borgeat%20A%5BAuthor%5D&cauthor=true&cauthor_uid=18070783). Sedation and regional anaesthesia in the adult patient. [Br J Anaesth.](http://www.ncbi.nlm.nih.gov/pubmed/?term=(ref%2C+BJA+100+(1)%3A+8-16).) 2008 Jan;100(1):8-16. 7. [Ekin A](http://www.ncbi.nlm.nih.gov/pubmed/?term=Ekin%20A%5BAuthor%5D&cauthor=true&cauthor_uid=24263045)1, [Donmez F](http://www.ncbi.nlm.nih.gov/pubmed/?term=Donmez%20F%5BAuthor%5D&cauthor=true&cauthor_uid=24263045), [Taspinar V](http://www.ncbi.nlm.nih.gov/pubmed/?term=Taspinar%20V%5BAuthor%5D&cauthor=true&cauthor_uid=24263045), [Dikmen B](http://www.ncbi.nlm.nih.gov/pubmed/?term=Dikmen%20B%5BAuthor%5D&cauthor=true&cauthor_uid=24263045). Patient-controlled sedation in orthopedic surgery under regional anesthesia: a new approach in procedural sedation. [Braz J Anesthesiol.](http://www.ncbi.nlm.nih.gov/pubmed/24263045) 2013 Sep-Oct;63(5):410-4. doi: 10.1016/j.bjan.2012.07.012. | |

1.11 Dissemination of Results

**(a)** Will the results be published at the end of the project?

**(b)** Will a plain English summary of the project be made directly available to participants at the end of the project?

If *Yes*, give details of the type of report and how it will be made available.

If *No*, explain why not.

|  |
| --- |

1.12 Adverse or unforeseen events

What procedures are in place to manage, monitor and report adverse and unforeseen events? (e.g. involving participants, other researchers and/or the management of information etc.).

| The study will be undertaken in the operating theatre, patients will therefore be closely monitored. In the event of any discomfort, the simulation can be prematurely terminated and regular anaesthetist controlled sedation reinstituted. Side effects are expected to be rare and mild and limited to nausea far less in intensity compared to those experienced intraoperatively on a regular basis. Severe side effects such as respiratory depression are very unlikely to occur at any rate above what is usually seen intraoperatively, given patient control over sedation, regular BIS monitoring, and anaesthetist supervision at all times. Similar studies have been conducted in other institutions, none of which have demonstrated any cases of severe side effects.  If a serious adverse event occurred the HREC will be notified within 48 hours. |
| --- |

SECTION D: PARTICIPANTS

1.13 Number of participants

**(a)** Total number of participants in the project (at all sites combined)

50

**(b)** Total number of participants at this site

50

**(c)** If the project involves more than one participant group (e.g. control and experimental groups, or different focus groups), how many participants will be in each group?

| 25 patients receiving IVR treatment and PCA, 25 patients receiving PCA only |
| --- |

1.14 Type of participants

**(a)** What categories of participants will be involved? *(e.g. oncology patients, children, people with learning disabilities, staff members etc.)*

| Healthy volunteers undergoing knee and hip replacement surgery |
| --- |

**(b)** What will be the age range of participants (if applicable)?

| 18 years of age or older |
| --- |

**(c)** Are there any ethical issues associated with the inclusion or exclusion criteria?

| No |
| --- |

1.15 Recruitment

**(a)** Are participants being recruited?

 *Go to Section E*

If Yes, describe the recruitment procedure, specific to:

- The source of participants
- How participants will be identified
- How participants will be contacted and by whom (indicating if the person making initial contact has any relationship to participants)
- The method(s) by which information is provided to participants (e.g. verbally, information sheet, fliers, posters, etc.)
- The setting in which information is provided (e.g. over the telephone, in a clinic or doctor’s surgery, through the mail, etc.)

| Theatre lists will be assessed for suitable patients in the week leading up to surgery. Patients will either be contacted over the phone or in person on the day of the operation. The study outline will be offered to them and a written consent will be obtained.  Recovering patients in the ICU without significant medical impairment without impaired cognitive function will be approached by Dr. Peter Chan and the study outlined to them. If they are interested in participation then a written consent will be obtained.  Both IVR and control groups will be consented for the procedure and will be informed how to press the sedation button. In order to reduce bias, control group patients will not be informed that other group will be receiving IVR therapy. |
| --- |

**(b)** Will any follow-up procedures be used to improve the rate of participation?

If *Yes*, describe the procedures.

|  |
| --- |

**(c)** Will any dependent or unequal relationship exist between anyone involved in the recruitment and the potential participants (e.g. counsellor/client, teacher/student, doctor/patient, warder/prisoner, etc./)?

If *Yes*:

(i) What is the nature of the dependent or unequal relationship?

|  |
| --- |

(ii) What measures will be taken to minimise the impact of the participant’s dependency so that the voluntariness of their consent is not compromised?

|  |
| --- |

**(d)** Will any other dual relationship exist between any researcher and participants?
For example, will any of the researchers also be:

- colleagues of participants;
- head of the department where it is proposed to recruit participants and carry out the research?

If *Yes*:

(i) What is the nature of the dual relationship?

|  |
| --- |

(ii) How will ethical issues arising from the dual relationship be addressed?

|  |
| --- |

**(e)** Will reimbursement, payment or other offers be made to participants?

If Yes, provide details.

|  |
| --- |

1.20 Information to participants

**(a)** Will written information about the project be given to participants?

If *No*, give reasons.

|  |
| --- |

(b) Who will explain the project to participants and obtain formal consent?

| Principal researcher |
| --- |

1.21 Consent

**(a)** How will informed consent be obtained?

|  |
| --- |

1.22 Consequences of participation

**(a)** Is there any possibility of discomfort to participants?

If *Yes*, please describe.

| These goggles have been used in many different non-clinical settings and are frequently used as video gaming devices. The only side effect reported to date has been nausea, and this is typically when running simulations involving significant movement. This nausea is expected to be far less in intensity compared to nausea typically experienced in both the ICU and operating theatre on a regular basis. As the patients are stationary and the simulations not involving movement, the risk of nausea is very low. As patients are awake, they can express discomfort and the simulation can be terminated prematurely in the event of discomfort.  To date, this simulation has been trialled on 15 staff members outside of the clinical environment, and all have tolerated and enjoyed the experience without any significant nausea and other side effects.  Our pilot study on 10 HDU patients and 10 patients receiving regional anaesthesia demonstrated nausea in a single patient in each group, which settled with antiemetics and cessation of the therapy.  The Oculus goggles are freely available for purchase for use in video gaming, and are currently being trialled on Qantas long-haul flights without any significant discomfort. |
| --- |

**(b)** Are there any potential benefits to the participants?

| The purpose of utilizing these goggles is to provide patients with a realistic virtual world that provides calming effects. They are expected to relax the patient and hopefully minimize the amount of sedation and analgesia used. Anecdotal evidence suggests a reduction in heart rate and blood pressure on patients who have used the goggles. |
| --- |

1.23 Other ethical issues

Does the project present any other ethical issues with respect to participation? *(e.g. issues related to illegal activities; indigenous or other special community or cultural groups; risks to third parties; etc)*

| None |
| --- |

SECTION E: COLLECTION/USE/DISCLOSURE OF INFORMATION

Researchers have a legal as well as an ethical obligation to consider privacy issues. The following questions assist the researcher, the HREC and the institution to fulfil their obligations under State and Commonwealth privacy legislation.

1.24 Collection of information

**(a)** Does the project involve collection of information about individuals without their knowledge or consent?

**If Yes, Please complete and submit Section 3 of the Victorian Specific Module with your application concerning collection, use and/or disclosure of information -* [*'Victorian Specific Module' retrieved from the health.vic.gov.au document library*](http://docs.health.vic.gov.au/docs/doc/Victorian-Specific-Module) *ensuring that all national statement criteria for waiving consent as set out in Chapter 2.3.10(a)-(*i)

**(b)** What type of information will be collected? (*Tick as many as apply*)

**(c)** Will participants’ consent be sought to use the collected information for

****Please note: if seeking extended or unspecified consent then research data will need to be “banked” for use in future related or future unspecified research. Research projects involving the establishment of a databank cannot be submitted via the Low Risk Research pathway and will need to submitted to Human Research Ethics Committee-A (HREC-A) for full review.***

**(d)** Does the project involve the establishment of a databank*?

**(e)** Does the Participant Information and Consent Form explain:

| What information is being collected, and why it is being collected |   |
| --- | --- |
| The period for which the records relating to the participant will be kept |   |
| The form in which the data will be stored (i.e. please state whether it will be non-identifiable, re-identifiable due to coding, or identifiable) |   |
| The steps taken to ensure confidentiality and secure storage of data |   |
| How privacy and confidentiality will be protected in any publication of the information |   |
| The fact that the individual may access that information |   |
| The contact details (including name/position/telephone number) of the investigator for participants to contact in the event they have questions |   |
| The contact details of the Executive Officer Research Governance Unit (03 9231 3930) for participants questions or complaints |  |

If you answered “No” to any of these questions, give the reasons why this information has not been included in the Participant Information and Consent Form.

|  |
| --- |

1.25 Records

**(a)** Will records be accessed to collect information?

If yes, how many records will be sourced and what is the source (e.g. medical record, participant in person) and the type of information that will be collected, used or disclosed (e.g. date of birth, medical history, number of convictions, etc.) (Please copy the table below and repeat for each source)

| **Source:** Participants Medical Records at St Vincent's Hospital  **Number of records:** 50  **Type of information:** Standard information used to describe major organ function and hospital, vital signs and parameters, quantity and type of antiemetic medication and analgesia used, intensive care stays and hospital discharge destinations. |
| --- |

**(b)** Does the project involve assigning unique identifiers to enable the sharing of information with other institutions and/or organisations?

If *Yes*, give details of how this will be carried out in accordance with relevant Privacy Principles (e.g. HPP 7, VIPP 7 or NPP 7).

| Case Report Forms with a log of the participants’ names and unique study numbers will be kept, with access restricted to the site’s Principal Investigator and his/her delegates. Electronic information will be stored on secure servers where access is restricted by password to the site Principal Investigators and their delegates. It will be necessary to confirm details and check data in the CRF against the original medical record and therefore it must be possible to identify the correct records. |
| --- |

**(c)** Does the project involve trans-border (i.e. interstate or overseas) data flow?

If *Yes*, give details of how this will be carried out in accordance with relevant Privacy Principles (e.g. HPP 9, VIPP 9 or NPP 9).

|  |
| --- |

**(d)** For what period of time will the information be retained? How will the information be disposed of at the end of this period?

| Data will be held until 15 years following the completion of the project. Information will be disposed securely: paper documents will be shredded and computerised data will be permanently erased so that it cannot be retrieved. |
| --- |

**(e)** Describe the security arrangements for storage of the information. Where will the information be stored? Who will have access to the information?

| Paper records, which will include identifiers, will be stored at the participating centres in secure, locked offices with access limited to the site Principal Investigators and his/her delegates. Electronic information at participating centres will be stored on secure servers where access is restricted by password to the site Principal Investigators and his/her delegates. |
| --- |

**(f)** How will the privacy of individuals be respected in any publication arising from this project?

| Only pooled data would be presented in any publication arising from this study. No individual will be identifiable. |
| --- |

SECTION F: FINANCIAL AND RELATED ISSUES

1.26 Indirect costs

Is funding available for any indirect costs associated with this project (e.g. conference / travel, recruitment incentives, equipment)?

If yes, please provide details.

| This project has been approved for 7800 AUD from the St. Vincent's Research Endowment Fund and up to 33000 AUD from the St Vincent's Catalyst Innovation Fund. |
| --- |

1.27 Project budget

Please provide a detailed project budget for review, including a breakdown of:

If a detailed budget is not being provided, give reasons.

| Oculus headsets: 2X current generation + 2X next generation: 1800AUD  Mobile Oculus Laptops: Cost price from nVidia: 3300AUD  Noise Cancelling Headsets: 218AUD  IP fees, conference fees, travel grants, software license fees: 2000AUD  Programmer Costs: 150 man hours X 35 dollars an hour: 5250AUD  Principal investigator stipend: 4000  Total cost: 16568 AUD |
| --- |

1.28 Source of funding

How will this project be funded? List all sources of funds (*e.g. commercial sponsorship, grant, departmental funds etc.). If funded by the Department of Human Services, please specify the Branch providing the funding.*

| Source | Amount in $ | Status of Funds | |
| --- | --- | --- | --- |
|  |  | Application pending | Funds Available |
| Research Endowment Fund | 7800 |  | Yes |
| Catalyst Innovation Fund | 33000 |  | Yes |

1.29 Funds coverage

Do the funds presently available or applied for cover all requirements to conduct the project?

If *No*, explain how the shortfall will be made up or dealt with.

|  |
| --- |

1.30 Declaration – Principal Investigator

I hereby declare that I accept full responsibility for the conduct of this research project according to the principles of the *National Statement on Ethical Conduct in Human Research* (2007).

I declare that all researchers and other personnel involved in this project are appropriately qualified and experienced or will undergo appropriate training to fulfil their role in this project.

As Principal Investigator, I will ensure that:

- Annual Progress reports will be provided to the HREC on or before the due date
- A Final Report and a copy of any published material at the end of the research project will be provided to the HREC;
- The HREC will be notified in writing immediately if any change to the project is proposed, and approval is received before proceeding with the proposed change
- The HREC is notified in writing immediately if any adverse event occurs after the approval of the HREC has been obtained.

As principal researcher, I will take responsibility for the confidential maintenance of records for a minimum of 7 years after completion of the project (or 15 years in the case of clinical trials) or as otherwise required by the institution/approving HREC.

**Principal Researcher** *(print name):*

**Signature Date**

1.31 Declaration –Research Team

I/WE, the researcher(s) agree:

1. To only start this research project after obtaining final approval from the Institution’s Human Research Ethics Committee (HREC);
2. To conduct this research project in accordance with the protocols and procedures as approved by the HREC;
3. To only carry out this research project where adequate funding is available to enable the project to be carried out according to good research practice and in an ethical manner;
4. To provide additional information as requested by the HREC;
5. To maintain the confidentiality of all data collected from or about project participants;
6. To agree to an audit if requested by the HREC;
7. To only use data and any tissue samples collected for the study for which approval has been given;
8. To only grant access to data to authorised persons; and
9. To maintain security procedures for the protection of privacy, including (but not restricted to): removal of identifying information from data collection forms and computer files, storage of linkage codes in a locked cabinet and password control for access to identified data on computer files.

**I/We have read the NHMRC *National Statement on Ethical Conduct in Human Research* (2007) and will observe the principles set out in that document in addition to the *Declaration of Helsinki*, and ICH Good Clinical Practice.**

**Associate Researcher** *(print name):*

**Signature Date**

**Associate Researcher** *(print name):*

**Signature Date**

**Research Co-ordinator** *(print name):*

**Signature Date**

1.32 Declaration - Head of Department

As Head of Department I hereby declare that I have read the above research project, and support its conduction within my Department.

Name of Head of Department (or appropriate person): ………………………………………

Name of Department (or relevant section): ………………………………………

**Head of Department** *(print name):*

**Signature Date**

**Where a researcher is also Head of Department, certification must be sought from the person to whom the Head of Department is responsible. Researchers who are also Department Heads or Divisional Directors must not approve their own research on behalf of the Institution.*

1.33 Declaration – Supporting Departments (if applicable)

As Head of Department providing support to the above research project, I hereby declare that I have read the above research project, and have reached an agreement with the Principal Investigator to provide the required resources to support the conduction of the project.

**Head of Department** *(print name):*

**Signature Date**
