# Supplementary material for: Effects of immersive virtual reality therapy on intravenous patient-controlled sedation during orthopaedic surgery under regional anesthesia: A randomized controlled trial
Source: PLoS One. 2020 Feb 24;15(2):e0229320. doi: 10.1371/journal.pone.0229320 (PMC7039521; doi:10.1371/journal.pone.0229320)
Supplement: S1 Appendix — (PDF) [file pone.0229320.s004.pdf]

S1 Appendix: Datasheet with modified QoR-40

Name: ____________________ UR#: _________________ Age: _________ Gender: ____

Procedure:

Propofol use hour 1 ______ Demands Hour 1 ______

Propofol use hour 2 ______ Demands Hour 2 ______

Propofol use hour 3 ______ Demands Hour 3______

Total Propofol use____ Total Demands _____

Other sedatives used _____________________

Obstruction/Apnoea during case Y/N

	**Start**	**End**
**Comfort 1-5 (1 = never, 5 = all the time)**		
Able to breathe easily		
Have had a good sleep		
Feel rested		
**Emotions**		
Feeling of general well-being		
Feeling in control		
Feeling comfortable		
**Comfort/Symptoms**		
Nausea		
Vomiting		
Dry-retching		
Feeling restless		
Shaking or twitching		
Shivering		
Feeling too cold		
Feeling dizzy		
**Emotions (B)**		
Bad dreams		
Anxious		
Angry		
Depressed		
Alone		
**Patient Support**		
Confused		
**Pain**		
Moderate Pain		
Severe Pain		
Headache		
Muscle Pain		
Backache		
Sore throat		
Sore mouth		
